# Postoperative Intra-abdominal Abscess Following General Surgery: A Systematic Review of Risk Factors, Prevention, and the Role of Laparotomy

**DOI:** 10.7759/cureus.91100

**Published:** 2025-08-27

**Authors:** Mawada Taha, Mohamed K Abouelsadat, Mohamed Elfakharany, Abdelrahman Ibrahim, Ahmed M Abufouda, Nasratullah Barai, Jarallah H. J. Alkhazendar, Aliaa H Alkhazendar, Anwar Al-Kassar, Ahmed Khan

**Affiliations:** 1 General Surgery, The National Ribat University, Khartoum , SDN; 2 Vascular Surgery, Royal Free Hospital, London, GBR; 3 General Surgery, Health Education England, Yorkshire and The Humber Deanery, Leeds, GBR; 4 Trauma and Orthopedic, University Hospitals of North Midlands NHS Trust, Stoke-on-Tent, GBR; 5 Colorectal Surgery, Mansoura Teaching Hospitals, Mansoura, EGY; 6 General Surgery, Gaza European Hospital, Gaza, PSE; 7 General Surgery, Royal Medical Complex, Kabul, AFG; 8 General and Emergency Surgery, East and North Hertfordshire NHS Trust, Lister Hospital, Stevenage, GBR; 9 Surgery, The Islamic University of Gaza, Gaza, PSE; 10 Vascular Surgery, Countess of Chester Hospital, Chester, GBR; 11 Medicine, Elizabethtown College, Islamabad, PAK

**Keywords:** general surgery, intra-abdominal infection, laparotomy, postoperative abscess, prevention, risk factors, source control

## Abstract

Postoperative intra-abdominal abscess (PIAA) is a significant complication following general abdominal surgery, contributing to increased morbidity and mortality. This systematic review was conducted following Preferred Reporting Items for Systematic Reviews and Meta-Analyses (PRISMA) 2020 guidelines, with a comprehensive search of multiple databases including PubMed, Embase, Scopus, and the Cochrane Library up to July 2025. Studies were selected based on predefined PICO (Population, Intervention, Comparison, Outcome) criteria, focusing on adult patients undergoing abdominal surgery and assessing risk factors, prevention, and management strategies for PIAA. Five eligible studies involving 3,537 patients were included and analyzed. Key risk factors identified included perforated appendicitis, intraoperative contamination, elevated National Nosocomial Infections Surveillance (NNIS) scores, preoperative radiation, and splenectomy. Preventive measures such as targeted perioperative antibiotic prophylaxis and selective surgical drainage have demonstrated efficacy in reducing abscess incidence. Percutaneous drainage is generally preferred as the initial intervention due to its minimally invasive nature and favorable outcomes, while laparotomy remains necessary for complex abscesses involving multiloculated collections or anastomotic leaks. The routine use of prophylactic drains remains controversial and may increase postoperative complications. Effective management of PIAA requires careful risk assessment, individualized preventive strategies, and prompt source control. Further well-designed randomized controlled trials are needed to establish standardized treatment protocols and optimize patient outcomes.

## Introduction and background

Postoperative intra-abdominal abscess (PIAA) is a significant and potentially life-threatening complication that can occur after general abdominal surgery. It is characterized by a localized accumulation of pus within the peritoneal cavity, typically arising as a result of contamination, anastomotic failure, or insufficient source control during the initial surgical procedure [1]. The incidence of PIAA varies based on the type of surgical procedure performed, the patient’s underlying health conditions, and intraoperative circumstances, with reported rates ranging from 1% to 10% in large surgical series. It is estimated that up to 70% of intra-abdominal abscesses are postoperative in origin, with approximately 6% of patients undergoing colorectal procedures developing this complication. Hepatic abscesses make up about 13% of all intra-abdominal abscesses, with a predominance in the right hepatic lobe, likely due to its larger volume and more robust vascularization [2]. In the context of appendectomies, PIAA occurs in approximately 3% to 25% of cases, with the highest incidence observed in patients with complicated appendicitis [3]. If not promptly identified and managed, PIAA can lead to substantial morbidity and even mortality. Patients who develop PIAA often present with fever, leukocytosis, abdominal pain, and ileus, but symptoms can be subtle and nonspecific, making diagnosis difficult without imaging.

A delayed or missed diagnosis of PIAA can result in severe complications such as sepsis, extended hospital stays, readmissions, and the need for invasive treatments, including repeat surgical intervention. Several risk factors have been identified, including inflammatory bowel disease, emergency surgeries, malnutrition, corticosteroid therapy, and intraoperative contamination [4]. Although advancements in diagnostic imaging and the use of percutaneous drainage have significantly improved management outcomes, these methods are not universally effective, particularly in cases involving multiloculated abscesses or when anatomical access is restricted. In such scenarios, open surgical re-intervention through laparotomy is often required to achieve adequate source control [5]. Increasing attention has been given to prevention strategies, such as enhanced recovery after surgery (ERAS) protocols, early initiation of enteral feeding, intraoperative testing for anastomotic leaks, and selective application of surgical drains. Despite these efforts, the use of prophylactic drainage remains a topic of ongoing debate, with recent studies yielding inconclusive findings [6]. This systematic review aims to consolidate current evidence concerning risk factors, prevention measures, the effectiveness of prophylactic drainage, and the role of laparotomy in the management of PIAA following general abdominal surgery.

## Review

Materials and methods

Search Strategy

This systematic review adhered to the Preferred Reporting Items for Systematic Reviews and Meta‑Analyses (PRISMA) 2020 guidelines to ensure methodological rigor [7]. A comprehensive search was performed across four major biomedical databases, such as PubMed, Medline, Embase, Scopus, and the Cochrane Library, up to July 2025. The following search terms and Medical Subject Headings (MeSH) were included: “postoperative intra-abdominal abscess,” “general surgery,” “abdominal infection,” “anastomotic leak,” “risk factors,” “prophylactic drainage,” “surgical site infection,” “prevention,” and “laparotomy.” Boolean operators (AND, OR) were applied to enhance search sensitivity and specificity. The search was restricted to human studies published in English.

Eligibility Criteria

The criteria were established based on the PICO (Population, Intervention, Comparison, Outcome) framework [8], eligible studies included adult patients (≥18 years) undergoing general abdominal surgery (Population), examining risk factors or preventive strategies for PIAA (Intervention), compared with patients without abscess or those managed with alternative approaches (Comparator), and reporting on abscess incidence, prevention, or laparotomy outcomes (Outcomes). Studies were included if they (1) involved human adults, (2) focused on PIAA after surgery, (3) assessed risk factors or prevention, (4) evaluated laparotomy’s role, and (5) were published in English with full text. Exclusion criteria were (1) pediatric-only studies, (2) case reports or abstracts, (3) animal/lab-based studies, and (4) studies lacking defined clinical outcomes.

Study Selection

Two independent reviewers conducted an initial screening of titles and abstracts to assess their relevance to the research question. Studies that appeared to meet the inclusion criteria underwent full-text review for final eligibility assessment. Any disagreements between reviewers were resolved through consensus or, when necessary, by involving a third reviewer. The study selection process was systematically documented using a PRISMA flow diagram to ensure transparency and methodological rigor.

Data Extraction

Two reviewers independently extracted data from the included studies using a predefined and standardized format. Extracted information included the first author’s name, year of publication, study design, sample size, patient population, type of surgery performed, identified risk factors for PIAA, preventive strategies implemented, and clinical outcomes such as abscess incidence, need for laparotomy, morbidity, and mortality. Any discrepancies in data extraction were resolved through discussion or, when necessary, consultation with a third reviewer. Data were collected as reported in the original articles without assumptions or estimations.

Risk-of-Bias Assessment

Risk of bias was evaluated using the Risk Of Bias In Non-randomized Studies - of Interventions (ROBINS-I) tool for non-randomized studies [9] and the Newcastle-Ottawa Scale (NOS) for observational cohort studies [10]. Each study was assessed independently by two reviewers, and any discrepancies were resolved through discussion and consensus.

Data Synthesis

Due to variability in study designs, patient populations, and outcome measures across the included studies, a meta-analysis was not feasible; therefore, a narrative synthesis was conducted. Findings were grouped into three main categories: incidence and risk factors, preventive strategies, and management approaches, including the role of laparotomy. Where comparative data were available, outcomes such as abscess formation rates, postoperative complications, duration of hospitalization, and success rates of conservative versus surgical management were summarized qualitatively to highlight key trends and clinical implications.

Results

Study Selection Process

**Figure 1:**
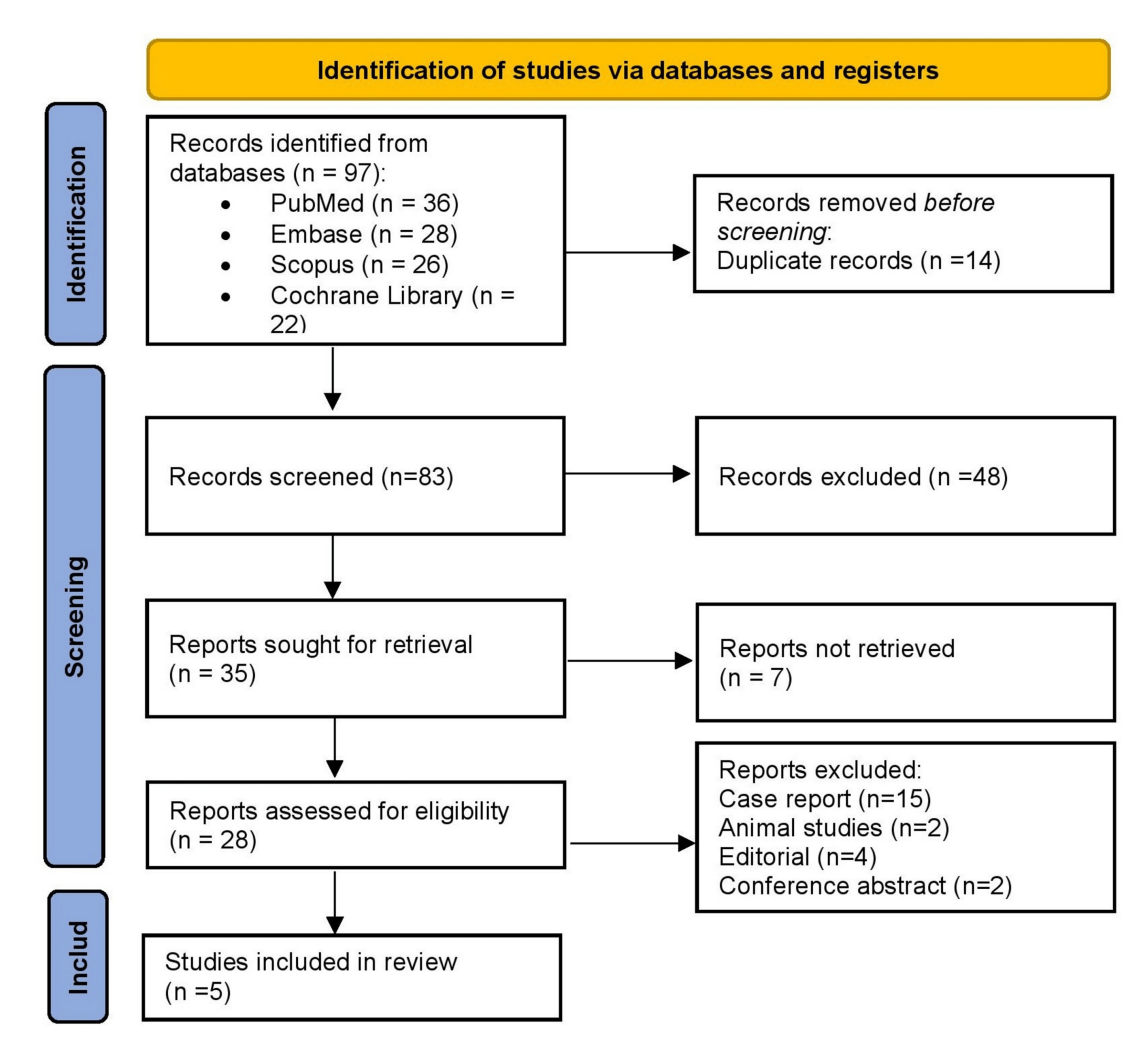
PRISMA 2020 flow diagram for the study selection process. PRISMA, Preferred Reporting Items for Systematic Reviews and Meta-Analyses

As illustrated in Figure 1, a total of 97 records were identified through systematic searches of four major databases: PubMed (*n* = 36), Embase (*n* = 28), Scopus (*n* = 26), and the Cochrane Library (*n* = 22). After removing 14 duplicate entries, 83 unique records remained for title and abstract screening. Based on initial screening, 48 records were excluded for not meeting the inclusion criteria. The full texts of 35 articles were assessed for eligibility. Of these, 7 reports could not be retrieved due to access limitations. The remaining 28 full-text articles were reviewed in detail, leading to the exclusion of 23 studies for the following reasons: case reports (*n* = 15), animal studies (*n* = 2), editorials (*n* = 4), and conference abstracts (*n* = 2). Ultimately, five studies fulfilled all eligibility criteria and were included in the final systematic review.

Characteristics of the Selected Studies

Table 1 summarizes five studies involving approximately 3,537 patients on postoperative intra-abdominal abscess (PIAA) after abdominal surgeries. Son et al. [11] studied 144 children with perforated appendicitis, identifying key risk factors for abscess after laparoscopic appendectomy. Cho et al. [12] analyzed 1,817 adults and found that peritoneal irrigation without drainage increased abscess risk. Reid et al. [13] reported higher abscess rates in 417 adults with perforated appendicitis after open surgery. Barmparas et al. [14] noted increased abscess incidence in 33 splenectomy patients out of 526 abdominal surgeries. Kobayashi et al.[15] linked high surgical risk and radiation to abscesses in 633 colorectal surgery patients. These studies highlight important factors affecting abscess formation and guide postoperative care.

**Table 1:** Characteristics of the selected studies. P, population; I, intervention/exposure/condition; C, comparator; O, outcomes; IAA, intra-abdominal abscess; CRP, C-reactive protein; US, ultrasound; CT, computed tomography; RLQ, right lower quadrant; OR, odds ratio; NNIS, National Nosocomial Infections Surveillance

Authors and year	Population (P)	Exposure/condition (I)	Comparator (C)	Outcomes (O)	Pathophysiological findings	Anatomical impact
Son et al. (2024)[11]	Children (<19 years) with perforated appendicitis (*n* ≈ 144)	Laparoscopic appendectomy with/without drain	Developed vs. no IAA	Incidence of IAA, re-intervention, and hospital stay	Appendicolith, ↑ CRP, and longer symptoms linked to abscess	Pelvic/peritoneal collections seen on US/CT
Cho et al. (2015) [12]	Adults undergoing laparoscopic appendectomy (*n* ≈ 1,817)	Peritoneal irrigation without drain placement	IAA vs no IAA groups	IAA rate (~1.5%), risk from irrigation alone	A contaminated field without drainage increases risk	RLQ and pelvic abscess formations
Reid et al. (1999) [13]	Adults undergoing open appendectomy (*n* = 417)	Perforated/gangrenous appendix	Non-perforated appendicitis	Higher IAA with perforation/gangrene (7.5%)	Inflammatory necrosis contributed to the collection	Retrocecal or pelvic fluid loculation
Barmparas et al. (2015) [14]	Adults undergoing abdominal surgery incl. splenectomy (*n* = 526; splenectomy (*n* = 33)	Splenectomy vs other abdominal procedures	Splenectomy vs non-splenectomy	IAA rate (9% in splenectomy vs 3% in controls); adjusted OR 4.3	Altered immune defense and local hematoma may promote infection	Abscess formation in the splenic bed or the perisplenic region
Kobayashi et al. (2013) [15]	Adults undergoing elective colorectal surgery (*n* = 633)	NNIS risk index ≥2, pre-op radiation	Low-risk surgical group	Anastomotic leak (6.3%), post-op IAA, morbidity	High NNIS, radiation exposure linked to leakage and infection	Pelvic collections secondary to anastomotic failure

Risk-of-Bias Assessment

Table 2 summarizes the risk of bias across the five included studies, which were assessed using design-appropriate tools. The retrospective cohort studies by Son et al. [11], Cho et al. [12], and Kobayashi et al. [15] were evaluated using the Newcastle-Ottawa Scale (NOS), and each was rated as having a moderate risk of bias. These studies had well-defined outcomes and populations but were limited by potential confounding and inherent bias in retrospective designs. Reid et al. [13], also a retrospective study, was rated as high risk due to its small sample size, outdated methodology, and incomplete reporting. The prospective cohort study by Barmparas et al. [14] was assessed using the ROBINS-I tool and judged to have a low to moderate risk of bias, owing to its prospective design, use of adjusted statistical models, and clear outcome assessment, though some residual confounding remained. Overall, while limitations exist, the studies were methodologically sound enough to provide reliable evidence when interpreted cautiously.

**Table 2:** Risk of bias across included studies. NOS, Newcastle–Ottawa Scale; ROBINS-I, Risk Of Bias In Non-randomized Studies - of Interventions

Study	Study design	Risk-of-bias tool	Risk-of-bias rating	Justification
Son et al. (2024) [11]	Retrospective cohort	Newcastle-Ottawa Scale (NOS)	Moderate	Clear inclusion criteria and outcome definitions; some risk of selection bias and confounding due to the retrospective nature.
Cho et al. (2015) [12]	Retrospective cohort	Newcastle-Ottawa Scale (NOS)	Moderate	Large sample size; potential for measurement bias and unmeasured confounding; outcomes well defined.
Reid et al. (1999) [13]	Retrospective cohort	Newcastle-Ottawa Scale (NOS)	High	Older study with less standardized reporting, small sample size, potential selection bias, and incomplete outcome data.
Barmparas et al. (2015) [14]	Prospective cohort	ROBINS-I	Low to moderate	Prospective design reduces recall bias; some confounding is likely, but adjusted analyses are performed; clear outcome assessment.
Kobayashi et al. (2013) [15]	Retrospective cohort	Newcastle-Ottawa Scale (NOS)	Moderate	Well-defined population and outcomes; retrospective design with possible confounding; adjusted for major variables.

Discussion 

PIAA remains a significant complication following a variety of abdominal surgeries, including appendectomy, splenectomy, colorectal resections, hepatic and renal surgeries, as well as aortic reconstructions. The latest guidelines from the Surgical Infection Society (2024) emphasize the importance of targeted perioperative antibiotic prophylaxis, particularly in hepatobiliary and colorectal surgeries. Antibiotic regimens tailored to the microbial flora identified in bile or preoperative cultures, such as Piperacillin-Tazobactam, are now preferred over traditional cephalosporin, supported by strong evidence (Grade 1-A). In cases of complicated appendicitis, short postoperative courses (24-48 hours) of either oral or intravenous antibiotics have demonstrated efficacy comparable to longer regimens, resulting in fewer complications and shorter hospital stays [16]. The risk of abscess formation is notably increased in contaminated surgical fields.

Specifically, Son et al. [11] identified that in pediatric patients with perforated appendicitis, the presence of an appendicolith, elevated C-reactive protein (CRP) levels, and delayed clinical presentation significantly heighten the risk of developing PIAA. In adults, Cho et al. [12] observed that performing peritoneal irrigation without subsequent drainage raises the likelihood of abscess formation. Similarly, Reid et al. [13] reported that open procedures involving perforated or gangrenous appendicitis are associated with an intra-abdominal abscess incidence as high as 7.5%. Among splenectomy patients, Barmparas et al. [14] noted an approximately threefold increase in abscess rates, possibly attributable to local hematoma formation and impaired immune response. Furthermore, Kobayashi et al. [15] demonstrated that elevated National Nosocomial Infections Surveillance (NNIS) risk scores and preoperative radiation therapy are strong predictors of anastomotic leaks and subsequent pelvic abscess development following colorectal surgery.

When abscesses do develop, percutaneous drainage is generally regarded as the first-line intervention. Numerous studies and systematic reviews have shown that percutaneous drainage yields morbidity and mortality outcomes comparable to open surgical drainage but offers advantages such as reduced hospital stay, lower costs, and less invasiveness. For instance, a prospective study involving 39 postoperative abscess cases revealed equivalent success rates between percutaneous and surgical drainage, with a preference for the former due to its minimally invasive nature [17]. Larger population analyses have further confirmed that percutaneous drainage is associated with decreased mortality rates (4.2% vs. 14.6%) and reduced intensive care admissions. Nonetheless, open surgical drainage and laparotomy remain indispensable in complex situations, including cases with multiple or loculated abscesses, anastomotic leaks, ischemic or necrotic tissue, or fecal contamination, where nonoperative approaches may be insufficient [18,19].

The current consensus favors on-demand laparotomy rather than planned re-laparotomies at fixed intervals, as the latter have not demonstrated a survival benefit and tend to increase healthcare resource utilization. Additionally, open abdomen techniques such as laparotomy with Bogota bag closure are reserved for select high-risk patients experiencing refractory sepsis or abdominal compartment syndrome, given their resource-intensive nature and potential complications. Regarding the use of drains during the initial surgery, evidence supports selective placement rather than routine prophylactic drainage. Both Cho et al. [12] and Son et al. [11] found that peritoneal irrigation without drainage increases the risk of abscess formation, while indiscriminate use of drains may lead to adverse effects such as ileus and bowel obstruction, especially in pediatric populations. Therefore, drains should be reserved for cases with evident contamination or high risk of leakage and avoided in clean surgical fields to minimize harm.

In summary, PIAA development is influenced by multiple factors, including patient comorbidities, surgical contamination, and intraoperative management. Effective prevention strategies encompass thorough risk assessment, individualized antibiotic prophylaxis, judicious use of surgical drains, and prompt, appropriate source control. Percutaneous drainage should be the preferred initial treatment whenever feasible, with laparotomy reserved for refractory or anatomically challenging cases. Adhering to these evidence-based principles can reduce PIAA incidence and enhance postoperative outcomes across diverse abdominal surgeries. Despite extensive research, the heterogeneity of study designs, patient populations, and surgical procedures introduces variability in reported outcomes. Additionally, the optimal duration and choice of antibiotic therapy remain subjects of ongoing investigation. The role of prophylactic drainage, although better defined, still lacks universal consensus, especially in complex or high-risk surgeries. Future randomized controlled trials with standardized protocols are needed to refine management algorithms and further clarify the indications for invasive interventions.

## Conclusions

PIAA remains a common complication after abdominal surgeries, particularly in contaminated or high-risk cases. Timely antibiotic therapy, selective use of drainage, and prompt source control are crucial to improving outcomes. Percutaneous drainage is effective for most abscesses, while laparotomy should be reserved for complex cases. It is suggested that standardized protocols for risk assessment and tailored interventions be developed to further reduce incidence and improve patient care.

## References

[1] Mehta NY, Marietta M, Copelin Copelin (2025). Intraabominal Abscesses. https://www.ncbi.nlm.nih.gov/books/NBK519573/.

[2] Bassetti M, Eckmann C, Giacobbe DR, Sartelli M, Montravers P (2020). Post-operative abdominal infections: epidemiology, operational definitions, and outcomes. Intensive Care Med.

[3] Mulita F, Plachouri KM, Liolis E, Kehagias D, Kehagias I (2021). Comparison of intra-abdominal abscess formation after laparoscopic and open appendectomy for complicated and uncomplicated appendicitis: a retrospective study. Wideochir Inne Tech Maloinwazyjne.

[4] Huang W, Tang Y, Nong L, Sun Y (2015). Risk factors for postoperative intra-abdominal septic complications after surgery in Crohn's disease: a meta-analysis of observational studies. J Crohns Colitis.

[5] Weiser TG, Forrester JD, Forrester JA (2019). Tactics to prevent intra-abdominal infections in general surgery. Surg Infect (Larchmt).

[6] Rekavari SG, Mahakalkar C (2024). Prophylactic intra-abdominal drains in major elective surgeries: a comprehensive review. Cureus.

[7] Page MJ, McKenzie JE, Bossuyt PM (2021). The PRISMA 2020 statement: an updated guideline for reporting systematic reviews. BMJ.

[8] Brown D (2020). A review of the PubMed PICO tool: using evidence-based practice in health education. Health Promot Pract.

[9] Sterne JA, Hernán MA, Reeves BC (2016). ROBINS-I: a tool for assessing risk of bias in non-randomised studies of interventions. BMJ.

[10] Lo CK, Mertz D, Loeb M (2014). Newcastle-Ottawa Scale: comparing reviewers' to authors' assessments. BMC Med Res Methodol.

[11] Son J, Han JW, Oh C (2024). Risk factors for postoperative intra-abdominal abscess in pediatric perforated appendicitis following laparoscopic appendectomy: a multicenter analysis. Children (Basel).

[12] Cho J, Park I, Lee D, Sung K, Baek J, Lee J (2015). Risk factors for postoperative intra-abdominal abscess after laparoscopic appendectomy: analysis of 1,817 consecutive cases. Dig Surg.

[13] Reid RI, Dobbs BR, Frizelle FA (1999). Risk factors for post-appendicectomy intra-abdominal abscess. Aust N Z J Surg.

[14] Barmparas G, Lamb AW, Lee D, Nguyen B, Eng J, Bloom MB, Ley EJ (2015). Postoperative infection risk after splenectomy: a prospective cohort study. Int J Surg.

[15] Kobayashi M, Mohri Y, Ohi M, Inoue Y, Araki T, Okita Y, Kusunoki M (2014). Risk factors for anastomotic leakage and favorable antimicrobial treatment as empirical therapy for intra-abdominal infection in patients undergoing colorectal surgery. Surg Today.

[16] Huston JM, Barie PS, Dellinger EP (2024). The Surgical Infection Society guidelines on the management of intra-abdominal infection: 2024 update. Surg Infect (Larchmt).

[17] Bufalari A, Giustozzi G, Moggi L (1996). Postoperative intraabdominal abscesses: percutaneous versus surgical treatment. Acta Chir Belg.

[18] Hemming A, Davis NL, Robins RE (1991). Surgical versus percutaneous drainage of intra-abdominal abscesses. Am J Surg.

[19] Ukweh ON, Alswang JM, Iya-Benson JN (2023). Comparative analysis of percutaneous drainage versus operative drainage of intra-abdominal abscesses in a resource-limited setting: the Tanzanian experience. Ann Glob Health.

